# Combining single-molecule and expansion microscopy in fission yeast to visualize protein structures at the nanostructural level

**DOI:** 10.1098/rsob.230414

**Published:** 2024-02-07

**Authors:** Ilijana Vojnovic, Oliver D. Caspari, Mehmet Ali Hoşkan, Ulrike Endesfelder

**Affiliations:** ^1^ Department of Systems and Synthetic Microbiology, Max Planck Institute for Terrestrial Microbiology and LOEWE Center for Synthetic Microbiology (SYNMIKRO), Marburg, Germany; ^2^ Department of Physics, Carnegie Mellon University, Pittsburgh, PA, USA; ^3^ Department of Microbiology, Institute Pasteur, Paris, France

**Keywords:** single-molecule localization microscopy, photoactivated localization microscopy, expansion microscopy, *Schizosaccharomyces pombe*, protein retention yield, correlative expansion microscopy

## Abstract

In this work, we have developed an expansion microscopy (ExM) protocol that combines ExM with photoactivated localization microscopy (ExPALM) for yeast cell imaging, and report a robust protocol for single-molecule and expansion microscopy of fission yeast, abbreviated as SExY. Our optimized SExY protocol retains about 50% of the fluorescent protein signal, doubling the amount obtained compared to the original protein retention ExM (proExM) protocol. It allows for a fivefold, highly isotropic expansion of fission yeast cells, which we carefully controlled while optimizing protein yield. We demonstrate the SExY method on several exemplary molecular targets and explicitly introduce low-abundant protein targets (e.g. nuclear proteins such as cbp1 and mis16, and the centromere-specific histone protein cnp1). The SExY protocol optimizations increasing protein yield could be beneficial for many studies, when targeting low abundance proteins, or for studies that rely on genetic labelling for various reasons (e.g. for proteins that cannot be easily targeted by extrinsic staining or in case artefacts introduced by unspecific staining interfere with data quality).

## Graphical abstract

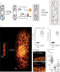

## 1. Introduction

The resolution of optical microscopy is constrained by the diffraction limit of light at about 200 nm [1]. However, most of molecular organization occurs in dimensions below this limit and therefore cannot be resolved by conventional light microscopic techniques. Super-resolution fluorescence microscopy (SRM) can be used to circumvent the diffraction limit by modulating the fluorescence of fluorescently labelled molecules at a sub-diffraction level, thereby discriminating between them [2]. This is achieved either by precisely defined illumination patterns (e.g. in STED [3] and SIM [4] imaging) or by single-molecule localization microscopy (SMLM) methods, in which the fluorescence of individual, on- and off-blinking or on- and off-binding fluorophores is separated in time (e.g. in PALM [5], (d)STORM [6,7] or PAINT [8] techniques). More recently, expansion microscopy (ExM) techniques have been developed that physically expand the biological sample, increasing resolution by practically a factor of 3–20-fold as a result of inflating the structures [9–13]. In contrast to the ‘classical' SRM methods, where preparing a good sample mostly relies on choosing the right fluorophores (e.g. bright (all) and either photostable (SIM, STED) or well-controlled in photoswitching (SMLM)) and optimizing for efficient and specific labelling (e.g. small labels for dense labelling, non-sticking labels for high specificity or genetic, covalent or high affinity labels for high efficiency) [2], ExM poses additional demands to the sample preparation due to the desired physical expansion: in ExM, the sample is anchored to a gel mesh and expanded upon incubation in aqueous media [9]. To achieve an isotropic expansion of the sample and thus a preservation of the underlying biological ultrastructure, all physical connections within the structure must be efficiently removed by, e.g. cell wall removal [14–17] and protein digestion steps [10,18–21], and all target molecules must be properly linked to the gel mesh before expansion by, for example, anchoring [22–24] or linkage by fixation [22]. In this context, implementing ExM in organisms with rigid cell walls [14–17,25–27] or maintaining isotropic expansion within samples of heterogeneous ‘rigidity' [20,28–31] is a particular challenge. Recent work has shown that samples can show different macro- and micro-expansion factors (e.g. factors that vary within a gel or tissue or differ between different organelles or domains within organelles [20,28–31]). Finally, protocols are often not directly transferable between different samples. For example, an ExM method that preserved isolated centrioles from *Chlamydomonas reinhardtii* failed to isotopically expand chromatin in root tips of barley [28,32].

A second challenge in ExM is to retain the molecular information of the sample while breaking physical connections for expansion. The protein retention yield is typically measured by how many target molecules can still be detected via fluorescent markers in an expanded sample [23]. It is mainly affected by the protocol steps of protein digestion and gelation. Protein digestion involves homogenization of the sample by cleavage of the proteins' peptide chains with proteinase K or collagenase type II, heat denaturation or homogenizing agents such as SDS [9,10,18,19,21]. Gelation involves the generation of radicals during the polymerization chain reaction of monomers to form a polyacrylamide gel (PAA). Only recently, a radical-free gelation method using novel custom-synthesized monomers was developed [33]. Both, protein digestion and gelation, thus have potential to reduce the protein retention yield by degrading target sites for staining or by degrading genetically encoded markers.

Protocols combining ExM with SRM techniques to date have achieved an overall resolution of 1–30 nm [34–37]. While a combination of ExM and SRM yields superior resolution, fluorophore choice and labelling strategy must suit both techniques, i.e. withstand and perform under the combined sample preparation and imaging protocols. To date, ExM was successfully combined with SIM [34], STED [35], dSTORM [36,37] and fluctuation-based techniques [38,39]. These ExM–SRM protocols rely on extrinsic labelling techniques of the targets using labels with organic dyes, mainly immunofluorescence [34–39]. Furthermore, dSTORM uses ionic switching buffers needed for the on- and off-blinking of dyes. These buffers however shrink expanded gels from deionized water. Thus, ExSTORM relies on expansion in ionic buffers, which achieves only lower expansion of about threefold [36]. An alternative is Ex-SMLM [37]. Here, gels expanded in deionized water are embedded in an uncharged secondary gel. These double-layer gels achieve an about threefold increase and tolerate incubation in ionic switching buffers.

While current ExM–SRM protocols work well for abundant targets and larger (polymeric) structures in various samples, staining background signal from non-specific adherent labels severely compromises imaging of sparse targets. Additionally, factors that hinder efficient extrinsic labelling, such as a cell wall or a highly crowded and charged cytosol like in microbial organisms, can complicate labelling [40] and can introduce artefacts [41]. To date, there are only a handful of publications on combined ExM and SRM strategies for microbiology, namely two studies using ExSIM of fungi [16] and bacteria [42], and one has combined ExM with SRRF imaging for viral SARS-Cov-2 particles [38].

In this work, we set out to establish a protocol for combined ExM and SMLM imaging in yeast. Here, our SMLM method of choice was PALM microscopy. In contrast to dSTORM, PALM microscopy makes use of genetic labelling using fluorescent proteins (FP) [2]. Thus, PALM samples intrinsically possess high labelling efficiency and specificity and do not rely on switching buffers which makes an implementation of ExPALM attractive for microbiological studies (at the price that current FPs offer lower photostability and photon yield compared to dyes). Nevertheless, as fluorescent proteins are affected and at least partially degraded by the ExM sample preparation protocols, so far, no protocols that combine ExM with PALM (ExPALM) exist.

Here, we combine PALM imaging with a proExM protocol [23] optimized for isotropy and protein retention for the fission yeast *Schizosaccharomyces pombe.* Using our optimized sample preparation protocols, we have established an ExPALM protocol that preserves about 50% of the FP signal, doubling the retained amount compared to applying the protein retention ExM (proExM) protocol to the tested *S. pombe* cell samples. Our optimized protocol achieves a fivefold, highly isotropic expansion of fission yeast cells which we tested for several molecular targets. Taken together, this protocol is the first demonstration of combined single-molecule and expansion microscopy of yeast (SExY microscopy).

## 2. Material and methods

### 2.1. Strain construction

C-terminal tagging of cbp1 with mEos2 [43] and sad1 with mScarlet-I [44] was adapted from [45]. First, two intermediate plasmids were created. For creating cbp1-mEos2, a pBluescript II SK+ plasmid containing the *Saccharomyces cerevisiae* ADH1 terminator, a kanamycin resistance gene and the mEos2 gene was constructed using primers with 20 bp overlap sequences (primers 9–14; electronic supplementary material, table S1) [45]. Similarly, for creating sad1-mScarlet-I, a pFA6-mScarlet-I-ADH1-hphMX6 plasmid was constructed amplifying mScarlet-I from pFA6a-mScarlet-I-hphNT1 [46] (primers 24–25) and pFA6-ADH1-hphMX6 (primers 15–16) from pFA6-hphMX6 [47]. DNA fragments were combined by Gibson Assembly (New England Biolabs, cat. no. E5510S), transformed into competent DH5*α* cells, streaked onto LB Amp^R^ plates and incubated overnight at 37 °C. Single colonies were picked, grown in LB Amp^R^ cultures for 2 h at 37 °C and plasmids were extracted (ZymoPure II Plasmid Midiprep kit, Zymo Research, cat. no. D4200S) and checked by sequencing (Eurofins Genomics Germany GmbH). In a second step, 200–600 bp DNA fragments up- and downstream of the respective insertion site were amplified from isolated genomic DNA from *h^−^ S. pombe* cells with 20 bp overlaps to the flanking genes using primers 1–4 for cbp1 and 17,19 & 22–23 for sad1. The DNA fragments were combined with the corresponding FP-ADH1-antibiotic resistance fragment using overlap-extension PCR [48] for sad1-mScarlet-I or SLiCE [49] for cbp1-mEos2. 10 µl of the PCR mix were transformed into either competent *h^+^ ade6-210 leu1-32 ura4-D18 mEos2:cnp1 or h- WT strain* using the Frozen-EZ Yeast Transformation II Kit (Zymo Research, cat. no. T2001). Cells were streaked onto YES agar plates, incubated overnight at 32 °C, replica plated onto either YES Kan^R^ or YES Hyg^R^ agar plates and grown at 32 °C for another two days. Successful genomic integration was checked by colony-PCR and DNA sequencing (Eurofins Genomics Germany GmbH).

For N-terminal tagging of mis16, DNA fragments up- and downstream of the insertion site where generated using primers 5–8. The DNA fragments were combined with mEos2-mis16 using SLiCE [49]. 10 µl of the PCR mix were transformed into either competent *h^+^ ade6-M210 leu1-32 ura4-D18 ura4nm41:mis16* cells using the Frozen-EZ Yeast Transformation II Kit (Zymo Research, cat. no. T2001). Cells were streaked out onto YES agar plates containing 1 g l^−1^ 5-Fluoroorotic Acid and 50 mg ml^−1^ uracil and grown at 32 °C for 2 days. Successful genomic integration was checked by colony-PCR and DNA sequencing (Eurofins Genomics Germany GmbH).

### 2.2. Protease enzyme activity assay

The colorimetric assay to measure protease activities was adapted from [50,51]. In short, casein (Sigma-Aldrich, cat. no. C7078-500G) was digested to release tyrosine. After stopping the digestion with tricholoroacetic acid (Sigma-Aldrich, cat. no. T0699-100ML), released tyrosine was measured by absorption at 660 nm using Folin & Ciocalteu's Phenol reagent (Sigma-Aldrich, cat. no. F9252-100ML). Enzymes were tested at their respective optimal working temperature and digestion buffer for 10 min. Concentration ranges were 0.2–1.0 U ml^−1^ for proteinase K, 0.2–1.0, 10 U ml^−1^ and 50 U ml^−1^ for Zymolyase and β-glucoronidase and 0.02–0.1 U ml^−1^ for lysing enzyme. Controls with 20 mM of the protease inhibitor phenylmethylsulfonylfluorid (PMSF, ThermoFisher Scientific, cat. no. 36978) were conducted for proteinase K and lysing enzyme and a standard curve of free l-tyrosine (Sigma-Aldrich, cat. no. T3754-50G) was measured.

### 2.3. *Schizosaccharomyces pombe* cell culture

*S. pombe* strains were grown on YES or EMM -leu agar plates at 32 °C for two days. Single colonies were picked and inoculated overnight in YES or EMM-leu medium at 25 °C. Overnight cultures were diluted to OD 0.05 for YES and OD 0.1 for EMM-leu cultures and grown at 25 °C to OD 0.5–0.8. For imaging non-expanded cells expressing cytosolic mEos2, the EMM-leu overnight cultures were diluted to OD 0.2 in EMM containing 5 µM thiamine to repress mEos2 expression and were grown for 3 h at 25 °C.

### 2.4. Sample preparation using the SExY protocol

The SExY protocol was adapted from the proExM method [23] and optimized for PALM and expansion microscopy in fission yeast. The ideal cell density for sample preparation was determined to be 6 OD ml^−1^ for all fluorescently-labelled protein of interest (POI) strains (electronic supplementary material, table S2) and 12 OD ml^−1^ for SP16 cells which express cytosolic mEos2 and were added to all samples for drift correction. The appropriate volumes were transferred to fresh Erlenmeyer flasks and fixed with 3.2% paraformaldehyde (Sigma-Aldrich, cat. no. F8775-4X25ML) and 0.1% glutaraldehyde (Sigma-Aldrich, cat. no. G5882-50ML) at 25 °C for 10 min. The cells were harvested by centrifugation (358*g*, 2 min, room temperature), resuspended in 1 ml 1x PBS and washed twice in 1 ml 1x PBS for 5 min. The cells were washed twice with 200 µl 1x S-c/pH 5.8 (1.2 M D-Sorbitol (Sigma-Aldrich, cat. no. S1876-500G) in citrate/phosphate buffer (Sigma-Aldrich, cat. no. C0759-1KG and cat. no. S7907-500G), pH 5.8) for 10 min each. The pellet after centrifugation was resuspended in 1 ml per 6 OD/ml Lallzyme MMX mix (100 mg ml^−1^ Lallzyme MMX (Lallemand, cat. no. ElO11-2240-15), 1.2 M d-Sorbitol in citrate/phosphate buffer, pH 5.8) and incubated at 30°C for 2 h for cell wall digestion. Subsequently, the cells were washed twice in 1 ml 1x S-PBS (1.2 M D-Sorbitol in 1x PBS, pH 7.4). To anchor the amine groups of the proteins to the PAA gel mesh, the cell pellets were resuspended in 200 µl per 6 OD ml^−1^ AcX solution (0.1 mg ml^−1^ Acryloyl X (Invitrogen, cat. no. A20770), 1% (v/v) DMSO (Carl Roth, cat. no. A994.1) in 1x S-PBS). Each POI strain was mixed with SP16 cells in a 1 : 2 ratio and incubated at 25 °C overnight. The next day, the sample was washed twice with 500 µl 1x S-PBS for 15 min. Round coverslips were cleaned in 1 M KOH for 30 min and rinsed with MilliQ water. Air-dried coverslips were incubated with 100 µl poly-l-lysine (Sigma-Aldrich, cat. no. P8920) for 20 min at room temperature (RT) and then assembled into custom made imaging gel cassettes made of polyoxymethylene (POM). Since a previous study found that an initial incubation of the monomer solution with the sample without the catalysator yielded higher expansion factors [31], the cell pellets were resuspended in half of the 1.06x Monomer solution (8.625% (w/w) Sodium Acrylate (Sigma-Aldrich, cat. no. 408220-25G), 2.5% (w/w) 37:1 Acrylamide : Bisacrylamide (Sigma-Aldrich, cat. no. A6050-100ML), 0.15% (w/w) *N*,*N*′-methylenebisacrylamide (Sigma-Aldrich, cat. no. M1533-25ML), 2 M NaCl (Sigma-Aldrich, cat. no. S3014-500G) in 1x PBS) together with 0.2% TEMED (Research Products Int, cat. no. T18000-0.05) and 0.05% of freshly prepared L-glutathione (Carl Roth, cat. no. 6832.3). This pre-gelation mix was pipetted onto the coverslip in the imaging gel cassette and incubated for 10 min at 37 °C in the dark. Gelation was initiated by addition of 0.2% APS (Carl Roth, cat. no. 9178.3) and the rest of the 1.06x Monomer solution to the pre-gelation mix and thoroughly mixed. The gel cassettes were placed into a humid environment using Petri dishes with wet paper towels wrapped in aluminium foil and incubated at 37 °C for 1 h. The gels were then incubated in 2 ml digestion buffer (50 mM Tris (Carl Roth, cat. no. AE15.2) pH 7.5, 1 mM EDTA (Sigma-Aldrich, cat. no. EDS-100G), 0.2% Triton X-100 (Sigma-Aldrich, cat. no. T8787-50ML), 1 M NaCl) with 3 U ml^−1^ effective proteinase K (ThermoFisher, cat. no. EO0491) at 37 °C for 30 min in the aluminium foil wrapped mini Petri dishes (Sarstedt, cat. no. 82.1135.500). Thereafter, gels were washed with MilliQ water, transferred to Petri dishes, wrapped in aluminium foil and fully expanded in 100 ml MilliQ water for 4 h at RT, exchanging MilliQ water every hour. Ibidi 8-well glass bottom slides (Ibidi, cat. no. 80807), which were previously cleaned with 1 M KOH (Sigma-Aldrich, cat. no. 221473-2.5KG-M) for 30 min and washed twice with MilliQ water, were incubated with poly-l-lysine for 20 min and air dried. The expanded gel was cut using a scalpel, transferred into an Ibidi well, excess water was removed and the gel was incubated for 10 min. After that, the gel was secured in place by embedding it in 2% agarose (Carl Roth, cat. no. 3810.3) and allowing the agarose to dry for 10 min. The gel was submerged in 300 µl MilliQ water and then imaged.

### 2.5. Tests of various sample preparation steps while optimizing the protocol

#### 2.5.1. Cell wall digestion

To lyse the cell wall, the chemically fixed cells were treated with various enzymes and tested for a homogeneous macroscale expansion via fluorescence microscopy. The used enzymes and conditions for cell wall digestion can be found in electronic supplementary material, table S3. All incubations were done agitating the samples in a thermo block.

#### 2.5.2. Permeabilization

For permeabilizing the cell membrane, the fixed and in 1xPBS washed cells were resuspended in 200 µl 1xPBS containing 0.1% Triton X-100 and incubated at 37 °C for 30 min. Afterwards the cell wall was digested and the initial proExM protocol for fission yeast was continued.

#### 2.5.3. Protein digestion by heat denaturation

The initial proExM protocol adapted for fission yeast was performed until the gelation step, where 0.05% glutathione were added to the gelation as described above. Afterwards, the gel was submerged into 2 ml of the 50 mM Tris.HCl buffer at pH 8.0 and incubated at 65°C for 15 min. Then the gel was incubated in the renaturing buffer (35 mM KCl_2_, 1 mM MgCl_2_, 1 mM DTT, 30% glycerol, 50 mM Tris.HCl, pH 8.0) at RT for 30 min.

#### 2.5.4. Protein digestion by homogenization with SDS and heat denaturation

The sample was prepared the same way as for the heat denaturation until protein digestion. The gel was then submerged in 2 ml freshly prepared SDS solution (200 mM SDS, 200 mM NaCl, 50 mM Tris.HCl, pH 8.0) and incubated at 37 °C for 30 min with a subsequent incubation at 65 °C for 15 min. After washing the gel with deionized water, it was fully submerged in renaturing buffer and incubated at RT for 30 min.

#### 2.5.5. Gelation including glutathione

To determine the effect of glutathione, 0–0.5% freshly prepared glutathione was added to the pre-gelation mix and incubated with the sample for 10 min at 37 °C in the dark. After initiation of the gelation, samples were taken for fluorescence intensity measurement, and the rest of the sample was digested and expanded as described above. The gel rigidity of the expanded gels was determined visually and haptically.

### 2.6. Microscope set-up

For all imaging experiments, a custom build set-up based on an automated Nikon Ti Eclipse microscopy body with suitable dichroic and emission filters (ET dapi/Fitc/cy3 dichroic, ZT405/488/561rpc rejection filter, ET525/50 for GFP and the green form of mEos2 or ET610/75 for mScarlet-I and the red photoconverted form of mEos2, all AHF Analysentechnik, Germany) and a CFI Apo TIRF 100 × oil objective (NA 1.49, Nikon) was used. A perfect focus system (Nikon, Germany) was used for *z*-axis control, except for ExPALM imaging, where the sample was imaged at 10–80 µm depth. All lasers (405 nm OBIS, 488 nm Sapphire LP and 561 nm OBIS, Coherent Inc. USA) were modulated by an acousto-optical tunable filter (AOTF; Gooch and Housego, USA). Fluorescence detection was performed by an emCCD camera (iXON Ultra 888, Andor, UK) at a pixel size of 129 nm. Image acquisition was controlled by a customized version of µManager [52].

### 2.7. Fluorescence imaging of GFP-nup132

Expanded and non-expanded GFP-nup132 cells were identified using a 488 nm laser (21 W cm^−2^ at the sample level). A fluorescence video of 50 frames with an exposure time of 100 ms per frame was acquired using 81 W cm^−2^ at the sample level.

### 2.8. Fluorescence imaging and analysis of sad1-mScarlet-I

While optimizing the ExPALM protocol, a sample was taken after every step of the protocol and placed on a cleaned and poly-l-lysine coated Ibidi 8-well glass bottom slide. After 15 min of settling time, the sample was either washed twice (1x PBS for fixation/permeabilization steps or 1x S-PBS for cell wall digestion/anchoring steps) or prevented from drying/shrinking by adding one to two drops of MilliQ water in gelation/expansion steps. Only cells in mitosis with two sad1-mScarlet-I spots were imaged. A video of 10 imaging frames of the sad1-mScarlet-I fluorescence was taken with an exposure time of 100 ms per frame and in epi-illumination using a 561 nm laser. Laser intensities as measured at the sample level were 3 W cm^−2^ (live-anchoring) and 6 W cm^−2^ (gelation-protein digestion). The integrated intensity of the fluorescent spots and a close-by background area were measured in a 14 × 22 pixel ROI in the first three imaging frames using ImageJ [53]. The final intensity measure was obtained by calculating the mean of the three frames for both ROIs and subtracting the background.

### 2.9. PALM imaging and analysis of expanded cells

Expanded cbp1-mEos2 and mis16-mEos2 cells were identified in the gel by their fluorescence in epi-illumination using the 488 nm laser (21 W cm^−2^ at the sample level) and PALM videos of 3000 - 5000 frames were acquired with an exposure time of 60–80 ms per frame photoconverting and imaging mEos2 by continuous illumination of 405 nm laser (3–12 W cm^−2^ at the sample level) and 561 nm laser (1.2 kW cm^−2^ at the sample level). For imaging the DNA, the DNA was stained after mEos2 read-out using 100 nM TO-PRO-3 Iodide (Invitrogen, cat. no. T3605) which was added to the imaging well on the microscope stage and incubated for 30 min. A fluorescence video of 50 frames with an exposure time of 100 ms per frame was acquired using the 561 nm laser (0.5 kW cm^−2^ at the sample level). Expanded mEos2-cnp1/sad1-mScarlet-I cells where identified by mScarlet-I fluorescence using the 561 nm laser. A fluorescence movie of 50 frames with an exposure time of 100 ms per frame was acquired using the 561 laser (0.5 kW cm^−2^ at the sample level). Remaining mScarlet-I fluorescence was photobleached (1.2 kW cm^−2^ at the sample level, 561 nm laser) and the mEos2 signal and the TO-PRO-3 Iodide DNA stain were imaged as described above.

All samples contained cells expressing cytosolic mEos2, which served as marker for drift correction. Each field of view contained at least one cell expressing cytosolic mEos2 and was drift-corrected by cross-correlation or by relative frame-to-frame shift (supplementary code and https://github.com/Endesfelder-Lab). Data with unreliable drift correction, which typically occurred at the end of PALM videos at low localization densities, were discarded.

Images were reconstructed in Rapidstorm 3.2 with a pixel size of 10 nm [54]. Localization precision was determined using the NeNA method [55,56]. For image representation, images were blurred by a Gaussian using their NeNA value as the sigma value. PALM images were overlaid with corresponding fluorescence images of sad1-mScarlet-I and DNA in ImageJ 1.52p.

### 2.10. PALM imaging and analysis of non-expanded cells

Cells expressing cytosolic mEos2 were fixed with 3.2% paraformaldehyde (Sigma-Aldrich, cat. no. F8775-4X25ML) and 0.1% glutaraldehyde (Sigma-Aldrich, cat. no. G5882-50ML) and embedded in 0.5% agarose gels in an Ibidi 8-well glass bottom slide together with a 1:500 dilution of sonicated, dark-red, 0.2 µm sized FluoSpheres (Molecular probes, cat. no. F8807). PALM videos of 3000 - 5000 frames were acquired with an exposure time of 60 ms per frame photoconverting and imaging mEos2 by pulsed illumination of 405 nm laser (3–12 W cm^−2^ at the sample level) and 561 nm laser (1.2 kW cm^−2^ at the sample level).

Each field of view was corrected by fiducial drift correction using a custom script (Python 3.7). Images were reconstructed in Rapidstorm 3.2 with a pixel size of 10 nm [54]. Localization precision was determined using the NeNA method [55,56]. For image representation, images were tracked in Rapidstorm 3.2 with an allowed blinking interval of five frames and a sigma set to the NeNA value. After filtering out all trajectories of three steps and less, a PALM image was reconstructed in Rapidstorm 3.2 and blurred by a Gaussian using the NeNA value as sigma value.

### 2.11. Determination of expansion factor

The expansion factor was measured by the cytosol width and the nuclear size of expanded and non-expanded cells. For the cytosol width, mean and standard error of cells in reconstructed PALM images of fixed and expanded cells expressing cytosolic mEos2 were measured. Raw SMLM data were used for this purpose, as cell peripheries appear sparser post-processing (e.g. due to combining multiple localizations of the same fluorescence event into one). For measuring the nuclear expansion factor, the mean and standard error of the nuclear diameter were determined in fluorescence images of expanded and non-expanded GFP-nup132 cells were taken. Cells with non-spherical nuclei were excluded from the analysis. The expansion factor was calculated by dividing the expanded cytosol width or nuclear size by the non-expanded cytosol width or nuclear size.

### 2.12. Assessment of microscale isotropy of individual expanded cells by evaluating the distribution of cytosolic mEos2 molecules

Nearest-neighbour distances between cytosolic mEos2 localizations in expanded cells were compared with cytosolic mEos2 localizations in non-expanded cells in R, v3.6.1 (https://www.r-project.org/), using RStudio, 2022.07.2 + 576 (https://www.rstudio.com/) and the spatstat package [57]. To be able to compare the distances, first, point densities (number of points per cell area) were evaluated for all cells. Next, for a given expanded cell, areas of the 30 non-expanded cells were enlarged *in silico* until point densities matched. Then, the average nearest-neighbour distances were calculated. Finally, the mean (*μ*) across non-expanded cells was subtracted from the value of the expanded cell (*x_i_*), and normalized to the standard deviation (*σ*) of the distribution of non-expanded cells: (*x_i_ − μ)/σ*. A two-sided Student's *t*-test was performed.

### 2.13. Data reproducibility statement

All experiments were performed at least in duplicate for negative results that did not lead anywhere further and at least in triplicate for forward-leading results from different days of sample preparation.

## 3. Results and discussion

**Figure 1.**
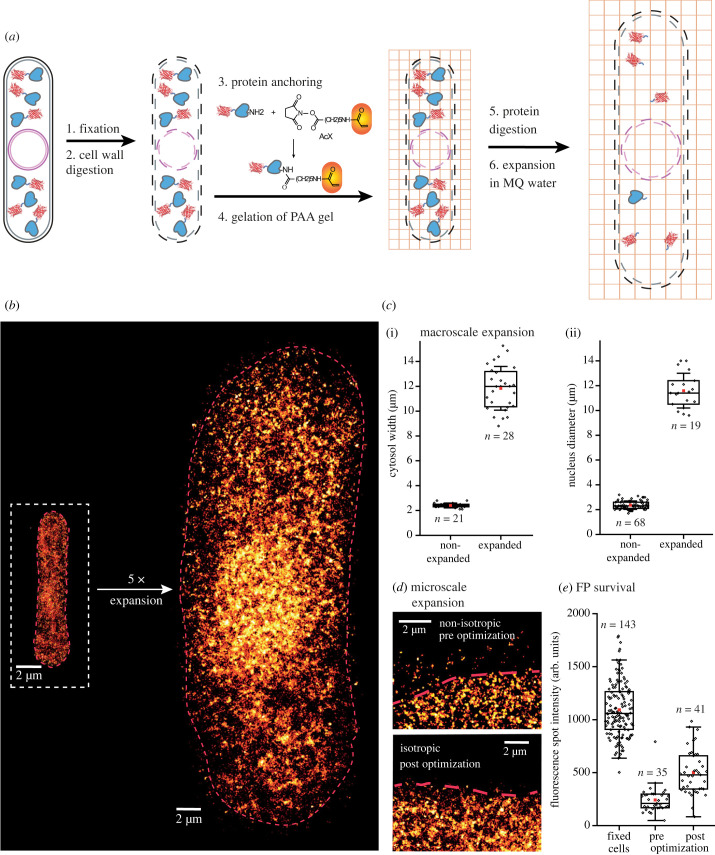
Principle and performance of the SExY method. (*a*) Principle of SExY sample preparation, a correlative ExPALM protocol for expanding yeast. Shown are cell wall (black), cell membrane (grey), inner and outer nuclear membrane (magenta), target protein (blue), FP marker (red) and the PAA gel mesh (orange). The acrylate group of the protein anchoring agent AcX is highlighted in orange. (*b*) Fission yeast cells expand approximately fivefold using the SExY protocol. (*c*) SExY provides a macroscale isotropic expansion as determined by two different samples, measuring (i) cytosol widths and (ii) nuclear diameter (Nup132) of non-expanded and expanded cells imaged over a total of nine different experiments. The expansion factor was determined to be 4.9 (s.e. 0.2, s.d. 0.8) (cytosol width) and 4.9 (s.e. 0.2, s.d. 0.9) (nuclear diameter), respectively. Note that nuclear widths were estimated from diffraction limited epifluorescence data and cannot be compared directly to cytosolic widths derived from SMLM data. (*d*) Additionally, the isotropic microscale expansion of the SExY protocol was probed investigating cell borders. Here, cells expanded with the SExY protocol show no mEos2 localizations being pulled outside of the cell area and hence an isotropic expansion on the microscale. (*e*) SExY retains 46% (s.e. 3.1%, s.d. 21.8%) of FP signal retention, whereas the original proExM protocol retained 22% (s.e. 2.0%, s.d. 12.4%).

In this work, we established a sample preparation and imaging protocol which combines the super-resolution technique PALM with a proExM protocol optimized for isotropy and protein retention for the fission yeast *S. pombe*, which we named Single-molecule and Expansion microscopy in fission Yeast (SExY). Our final protocol (figure 1*a*) consists of six steps: chemical fixation of the cells and subsequent cell wall digestion, protein anchoring and gelation of the PAA, which is then followed by protein digestion and finally expansion. SExY achieves an about fivefold expansion, as demonstrated by cells that cytosolically express the FP mEos2 whose positions were super-resolved by PALM imaging (figure 1*b*). While cytosolic, free diffusive mEos2 molecules are generally distributed throughout the cell, SExY also clearly resolves their (expected) non-random, small-scale substructured distribution (e.g. small omitted regions of round vesicles and a maximum of FPs near the nucleus).

Essential aspects of the SExY protocol are, first, an isotropic and satisfactory expansion, and second, a high protein retention yield to preserve protein yield (i.e. the FP signal for PALM read-out). Importantly, neither was the case when using the original proExM protocol on *S. pombe* cells. For verifying the isotropic expansion of the cells, we measured the cytosol width of cells expressing cytosolic mEos2 as seen in PALM images (figure 1*c*(i)). For verifying the isotropic expansion of organelles, we measured the nuclear diameter of cells in interphase that expressing GFP-tagged nuclear pore protein Nup132 (figure 1*c*(ii)). Non-expanded cells yielded a cytosol width of 2.4 µm ± 0.19 µm and a nuclear diameter of 2.36 µm ± 0.34 µm, cells expanded with the SExY protocol showed a cytosol width of 11.8 µm ± 1.8 µm and a nuclear diameter of 11.6 µm ± 1.4 µm. This resulted in a macroscale expansion factor of 4.9 (standard error (s.e.) 0.2, standard deviation (s.d.) 0.8) as measured by the cytosol width and a factor of 4.9 (s.e. 0.2, s.d. 0.9) for the nuclear diameter. Notably, the achieved expansion is higher than in current protocols that combine ExM with dSTORM and achieve an about threefold expansion [36,37]. To assess isotropic expansion on the microscale, we examined the cell boundaries of cells expressing cytosolic mEos2 which were expanded using either the initial proExM protocol (adding the step of using Lallzyme MMX for cell wall digestion) or the optimized SExY protocol. When applying the original protocol, we found a substantial fraction of mEos2 molecules located outside the main cell area within the polyacrylamide mesh network, apparently pulled outward due to heterogeneous expansion and resulting in ‘fuzzy' cell boundaries. By contrast, cell boundaries were clearly defined when the SExY protocol was applied (figure 1*d*), indicating a more defined and homogeneous microscale expansion. Furthermore, statistical testing for changes in cytosolic mEos2 FP distribution upon expansion did not show any changes (electronic supplementary material, figure S1). While prior to optimization of the protocol, individual expanded cells show a relatively high variation in cytosolic mEos2 distribution relative to non-expanded cells (up to 1.88 s.d.), post-optimization all expanded cells lie well within the range expected for isotropic expansion (up to 0.5 s.d. in relation to the mean of non-expanded cells).

To measure the protein retention yield, we quantified the fluorescent signal retained from the spindle pole protein sad1 tagged with mScarlet-I from cells during anaphase B in mitosis. For our initial proExM protocol, we found that only 22% (s.e. 2.0%, s.d. 12.4%) of the fluorescence signal was retained when compared to fixed cells (figure 1*e*). However, using the SExY protocol, about 46% (s.e. 3.1%, s.d. 21.8%) of the sad1-mScarlet-I signal was preserved, doubling the retained amount.

To gauge whether an increase in resolution could be achieved by expansion, localization precisions were estimated using the NeNA method [55] to be 13.9 nm (s.e. 0.8, s.d. 3.5, *n* = 22) for non-expanded cells and 26.5 nm (s.e. 0.7, s.d. 5.4, *n* = 55) for expanded cells (electronic supplementary material, figure S2). This implies a 1.9x (s.e. 0.1, s.d. 0.6) worse localization precision in SExY compared to conventional PALM. This change can be attributed to the reduced brightness of individual spots due to imaging in a larger depth of several micrometres to which our set-up is not adapted to. However, the 1.9x worse localization precision is compensated by the 4.9x expansion, which leaves us with an overall increase in optical resolution of 2.5x when going from conventional PALM imaging to SExY on our set-up. By using a set-up better suited for imaging at depth, this loss could be compensated further.

Altogether, these quality measures indicate that the SExY protocol is a promising approach to map protein structures in fission yeast. To achieve this, we have optimized the cell wall digestion, gelation and protein digestion steps in the development of the final SExY protocol. We report on these optimizations and discuss their rationale in detail in the following sections.

### 3.1. Efficient and complete cell wall digestion of fission yeast

**Figure 2.**
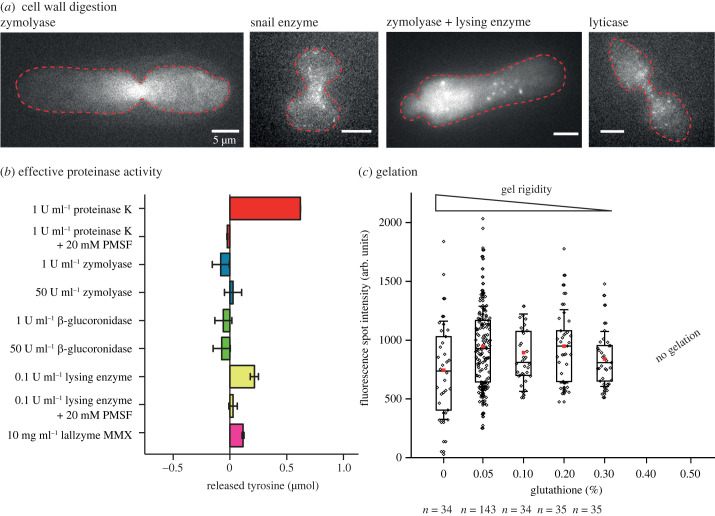
Protocol optimizations for SExY (*a*) Examples of incomplete cell wall digestion using zymolyase, snail enzyme, a combination of zymolyase and lysing enzyme and lyticase. All tested conditions lead to a partial and non-isotropic expansion constrained by remaining cell wall. (*b*) The proteinase activity of proteinase K (red) in comparison to different cell wall digesting enzymes measured by the release of tyrosine during casein digestion. Zymolyase (blue), β-glucoronidase (green), lysing enzyme (yellow) and Lallzyme MMX (magenta). Adding proteinase inhibitor PMSF successfully suppressed tyrosine release. (*c*) sad1-mScarlet-I signal shows an increase of 27% (s.e. 12.8%, s.d. 85%) in FP retention when adding 0.05% glutathione during gelation. Gel rigidity decreases with increasing glutathione concentration. Thus, no gel formation when adding 0.4% glutathione or higher was achieved.

We tested various cell wall removal enzymes commonly used in fission yeast studies [58–60] and visualized expanded *S. pombe* cells that cytosolically expressed mEos2. We found that zymolyase, zymolyase in combination with lysing enzyme, snail enzyme and lyticase caused partial expansion of yeast cells visible by ‘hour-glass' like shapes failing to expand the septum or earlier cytokinesis sites (figure 2*a*). Lallzyme MMX, chitinase and β-glucoronidase completely expanded *S. pombe* cells and showed comparable mEos2 signal to the non-expanded cells (figure 1*b*; electronic supplementary material, table S3).

The fact that we were able to fully expand fission yeast cells using a chitinase for cell wall digestion may indicate that chitin is present at the septum. The fission yeast septum consists of a primary septum composed predominantly of β-(1,3)-glucan and a flanking secondary septum composed of *α*(1,3)-glucan and branched *β*(1,6)-glucan [61,62]. Chitin synthase genes (chs1, chs2) are present, but their function is still controversial [63,64]. On the one hand, chs2 has been shown to be localized to the growing septal edges in vegetative *S. pombe* cells, yet chs2 possesses several mutations at sites critical for chitin synthesis [64–66]. We decided not to further investigate the ‘hourglass-like phenotypes' and the potential localization of chitin at the septum and instead proceeded to the next steps of protein digestion and gelation to be optimized, using Lallzyme MMX as our standard for cell wall digestion, as it was the cheapest and most reliable alternative among the three enzymes that resulted in complete expansion in our hands. In a recent study, *S. pombe* was successfully expanded using zymolyase for cell wall removal [67]. We speculate that this may be explained by the generally harsher conditions of this protocol, e.g. an additional fixation step with ice-cold acetone and replacement of proteinase K digestion with SDS treatment, which may be at the expense of protein retention yield, but this was not specified.

### 3.2. Increasing the protein retention yield

To evaluate how much fluorescent signal of the fluorescent proteins is retained after each step of sample preparation, we measured the fluorescent spot intensity of the mScarlet-I-tagged spindle pole protein sad1, whose structure and protein copy numbers is well defined during anaphase B in mitosis [68]. For our initial protocol, following the proExM protocol with an added step for cell wall digestion using Lallzyme MMX, only 22% (s.e. 2.0%, s.d. 12.4%) of the signal was retained compared to chemical fixed samples (figure 1*e*). This overall loss of fluorescent protein signal was mainly caused by three processing steps: A decrease of 18% (s.e. 2.8%, s.d. 29.6%) after permeabilization with 0.1% Triton X-100, a decrease of another 22% (s.e. 2.5%, s.d. 23.9%) after cell wall digestion using Lallzyme MMX compared to the previous step in the protocol and a decrease of another 46% (s.e. 2.0%, s.d. 12.4%) after protein digestion (electronic supplementary material, figure S3). We therefore targeted these three steps for optimization of the protocol.

In a first optimization, we excluded a separate permeabilization step using Triton X-100 as we hypothesized that the cell membrane is sufficiently disintegrated combining the steps of fixation with 3.2% paraformaldehyde and 0.1% glutaraldehyde and of protein digestion in a buffer containing Triton X-100. Cells expanded with a protocol excluding this step indeed expanded normally (examples in figure 1). Furthermore, we tested proteinase K as well as the different enzymes for cell wall removal for their proteinase activity in a colorimetric assay measuring tyrosine release in casein digestion (figure 2*b* and electronic supplementary material, figure S4). For proteinase K, we found that the proteinase activity for 1 U ml^−1^ of our stock was reduced to only 64% for freshly bought proteinase K and lower for older stocks. A control with protease inhibitor PMSF showed no residual proteinase activity. Therefore, for all further experiments, we determined the effective proteinase K activity using the colorimetric assay and adjusted the used concentrations accordingly. Among the cell wall removal enzymes, only the lysing enzyme and Lallzyme MMX showed proteinase activity, whereas zymolyase and β-glucoronidase showed no activity at 1 U ml^−1^ and even at higher concentrations of 50 U ml^−1^. Compared to proteinase K at a 10-fold higher concentration, lysing enzyme at a concentration of 0.1 U ml^−1^ showed rather strong activity. For Lallzyme MMX, we detected a (low) proteinase activity at 10 mg ml^−1^. Importantly, we found no significant decrease in fluorescence intensity of sad1-mScarlet-I after the exclusion of a separate permeabilization step with Triton X-100 (electronic supplementary material, figure S3b). We thus speculate that Lallzyme MMX without the detergent step at this early stage of the protocol might be hindered to freely access the cell, in particular the nucleus, and thus does not show visible proteinase activity on sad1-mScarlet-I fluorescence. When adjusting the proteinase K concentration for protein digestion (see below), we nevertheless could measure an effect when blocking the proteinase activity of Lallzyme MMX by PMSF. In general, different proteinase K activities in different laboratories could be one cause for inhomogeneous expansions [20,28–31].

On the basis of excluding a separate permeabilization step and using 10 mg ml^−1^ of Lallzyme MMX for cell wall digestion, we then optimized protein digestion and tested different methods that have been reported in the ExM literature: protein digestion using proteinase K [9], heat denaturation [10] and denaturation using SDS [19]. Homogenization by heat denaturation or treatment with SDS resulted in expanded fission yeast cells, but no or very low fluorescent signal for sad1-mScarlet-I or cytosolically expressed mEos2 was subsequently detected (data not shown). Using the proteinase K concentration from the proExM protocol, we were able to expand and image fluorescently labelled fission yeast, albeit with severe signal loss. As sufficient sample homogenization is critical for isotropic expansion, we tested different proteinase K concentrations to determine the minimal needed concentration of use. We found that the lowest effective concentration that allowed isotropic expansion on both, the macro- and the microscale of the sample was 3 U ml^−1^ (controlled for efficiency by the activity assay). Lower concentrations of proteinase K or a cell wall digest of Lallzyme MMX in the presence of the proteinase inhibitor PMSF followed by 3 U ml^−1^ proteinase K during protein digestion resulted in heterogeneous samples with a symptomatically increasing fraction of non-expanded cells cumulating at the bottom of the gel, only semi-expanded (two- to threefold expansion) or non-isotropic expanded cells, and only partially expanded gels (data not shown). For the final SExY protocol, we thus use the protease activity of 3 U ml^−1^ proteinase K combined with the protease activity of Lallzyme MMX which represents the minimum protein digestion level for homogeneous expansion of fission yeast to maintain a robust ExM protocol while retaining the maximum possible fluorescence signal.

In a final optimization, we then focused on the gelation step of the protocol as the free radicals that emerge during the gelation of the PAA gel have been shown to reduce the protein retention yield as well [23,33]. While we were not able to detect this loss in the initial protocol, we could detect the negative effect of the gelation process on protein retention after having optimized the other protocol steps (figure 2*c*). To minimize the loss, we tested whether a presumably milder gelation by adding the antioxidant glutathione (GSH), an efficient radical scavenger, would yield a higher retention yield, since glutathione can accept a radical at its sulfur moiety by hydrogen atom transfer (GS), adding a hydrogen at the former radical site of proteins [69,70]. To test this hypothesis, we measured the fluorescence intensity of sad1-mScarlet-I after PAA gelation for different glutathione concentrations and found a fluorescence increase of about 27% (s.e. 12.8%, s.d. 85%) when adding 0.05% glutathione (figure 2*c*). At higher glutathione concentrations, no further increase in fluorescence spot intensity but a decrease in PAA gel rigidity was observed until gelation was completely abolished at concentrations of 0.4% glutathione and higher (figure 2*c*). Presumably, the lower PAA gel rigidity stems from overall shorter PAA chains in the gel mesh, e.g. by glutathione reacting with growing PAA radical chains and thus terminating the chains early or by glutathione radicals (GS) forming glutathione disulfide (GSSG), decreasing the overall radical concentration. In the final SExY protocol, we thus use 0.05% glutathione during gelation.

**Figure 3.**
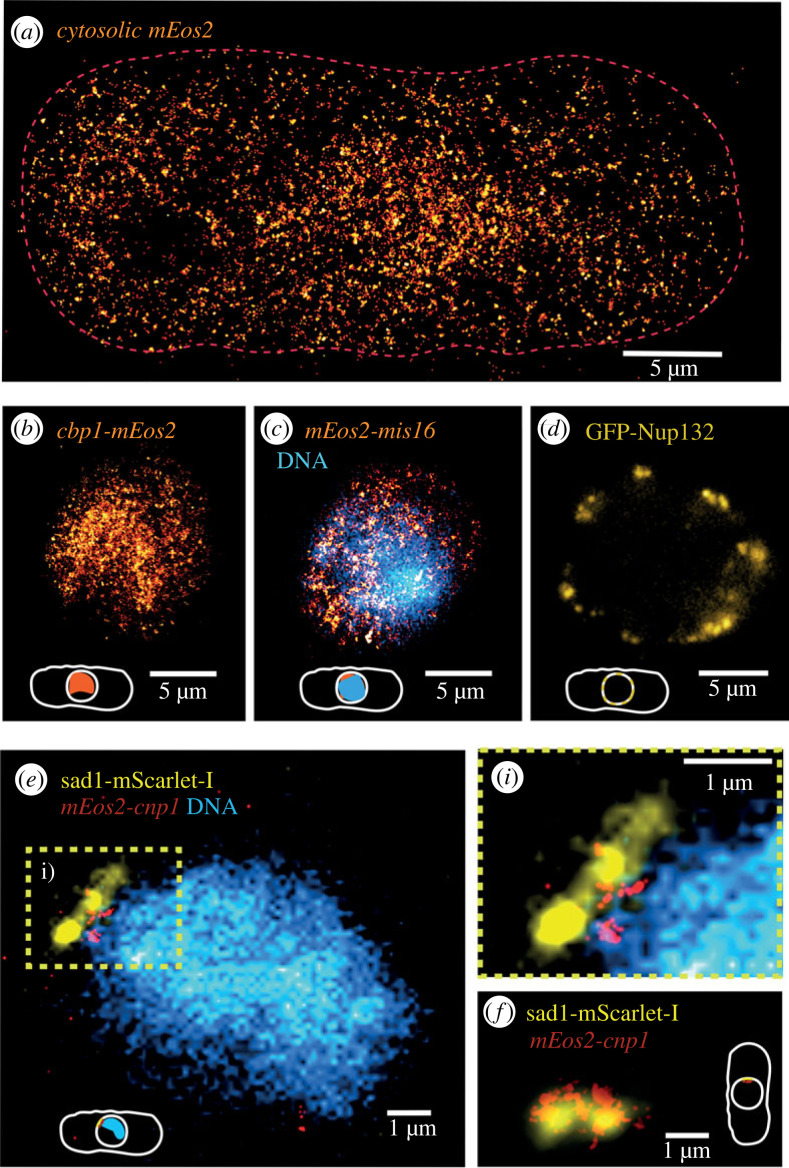
Examples of SExY microscopy SExY imaging of high (*a–c*) and low (*d–f*) abundant proteins in fission yeast. Super-resolved targets are marked in italic, targets imaged by conventional epifluorescence in regular script. (*a*) mEos2 expressed in the cytosol, (*b*) nuclear DNA binding protein cbp1, (*c*) nuclear protein mis16 and DNA (TO-PRO-3 Iodide), (*d*) nuclea pore protein Nup132, (*e,f*) centromeric histone protein cnp1 relative to the spindle pole body protein sad1; combined with visualizing DNA (TO-PRO-3 Iodide) (*e*, inset *i*)).

Finally, during this work, we tested several commonly used FPs for their fluorescence retention in ExM sample preparation as it is a known problem that chromophores often do not withstand treatments in correlative imaging protocols, e.g. when combining fluorescence microscopy with electron microscopy [71]. For conventional fluorophores, we tested GFP and mCherry, commonly used FPs of different origin from jellyfish and coral, in a dual-colour strain expressing two nuclear pore protein fusions [72]. While GFP-Nup132 was clearly visible, we could not detect mCherry-Nup131 in the expanded cells (figure 3*d*). Interestingly, we then found that mScarlet-I, a synthetic FP constructed by rational design based on several red FPs but with a high sequence identity of 86% to mCherry, survives the protocol and we could obtain images of expanded cells with mScarlet-I labelled spindle pole protein sad1 (figure 3*e,f*). Owing to mScarlet-I's superior brightness and red colour (allowing to image deeper into the samples than when using GFP), we decided to use it for the characterization of protein retention (figure 2) [73]. For PALM imaging, mainly FPs from Anthozoan corals, either DsRed-type like PAmCherry or Kaede-type like the Eos/Dendra/Maple FP family, are used [40]. We tested photoactivatable PAmCherry which showed no residual fluorescence already after fixation (data not shown). From previous studies, it is known that PAmCherry photoactivation is substantially reduced for fixation protocols including glutaraldehyde [74]. Within the ExM protocol, a comparably high glutaraldehyde concentration is used. We thus hypothesize that PAmCherry was fully quenched by glutaraldehyde when fixing the samples. By contrast, the green-to-red photoconvertible FP mEos2 survives the ExM protocol just fine. We therefore tagged different low and high abundant proteins with different cellular localizations with mEos2 to validate our imaging sensitivity and FP survival during SExY imaging. Using mEos2, we could successfully super-resolve proteins in expanded cells such as the nuclear DNA binding protein cbp1 (figure 3*b*) [75] and the nuclear protein mis16 (figure 3*c*) [76] as well as rather low-abundant proteins such as the centromere-specific histone protein cnp1 (figure 3*e,f*), which is only present in several tens to a few hundred copies per cell [41,77]. Using TO-PRO-3 Iodide, we co-visualized DNA (figure 3*c,e*).

On a small technical note, we also explored different drift correction strategies for PALM imaging of expanded gels. Drift correction of PALM data is commonly needed, as the recording of a PALM movie can take up to several minutes in which the sample can physically drift. This drift is often corrected by adding fiducial markers to the sample, e.g. fluorescent polystyrene beads or gold nanoparticles [78]. For expanded gels, we found that fiducial markers (even when covalently linking them to the gel mesh by amine groups) to a large extend either ended up at the bottom of the expanded gel and in case of polystyrene beads, high amounts of individual dyes leached out into the gel and resulted in cells coated by fuzzy highly fluorescent background signal (data not shown). Thus, we decided to use cross-correlation drift correction [79], which was previously also used in ExSTORM and Ex-SMLM [36,37]. While cross-correlation approaches work well for large statistics, thus directly on samples with high abundant proteins such as e.g. microtubules (as imaged in the ExSTORM and Ex-SMLM works), the correction of low-abundant protein signal fails due to insufficient statistics. Therefore, we mixed all samples with cells that cytosolically expressed mEos2 at high amounts and used the latter as markers for cross-correlation. Even at this for microbial PALM studies rather high level of several thousands of localizations per cell per movie, available cross-correlation approaches that cross-correlate sub-stacks of data fail to work reliably in many cases due to still too low statistics. Therefore, we implemented an additional drift correction method that determines the relative frame-to-frame shift between frames using tracked data of the cytosolically expressed mEos2 (supplementary code and https://github.com/Endesfelder-Lab).

## 4. Conclusion

With the combination of ExM with PALM (ExPALM), we close a gap in available super-resolution and ExM correlative protocols. We report the homogeneous expansion of fission yeast *S. pombe* cells to a fivefold expanded size while retaining about 50% of FP signal by several optimization steps of the original proExM protocol. The final protocol, which we termed SExY (Single-molecule and Expansion Microscopy of Yeast) is robust and reproducible, and extends the ExM toolbox by a correlative protocol for ExPALM imaging in microbiology. SExY explicitly excludes a separate permeabilization step with a detergent, uses Lallzyme MMX for digestion of the cell wall as an alternative to pricier enzymes and/or lysing components that only show heterogeneous or partial expansion (i.e. most striking ‘hour-glass' shaped phenotypes), and includes a fine-tuned protein digest using proteinase K at a controlled effective potency of 3 U ml^−1^ and a gentler PAA gelation by adding 0.05% glutathione. We suppose that the improved microscale isotropy in expansion as seen in SExY (figure 1*d*) stems from the dual effect of optimized homogeneous protein digestion and overall shorter PAA chains due to the milder gelation.

SMLM imaging has high demands for labelling specificity and efficiency. SExY as a correlative PALM imaging technique is especially suitable for low-abundant proteins as it does not rely on indirect target labelling via organic dyes using immunofluorescence and covalent bioconjugation methods (which are prone to unspecific staining due to the charge and molecular crowding in microbes [41]) and is optimized for a high protein retention yield retaining 50% of the FP signal. Cells expanded by the SExY protocol show a high fivefold expansion compared to Ex-SMLM and ExSTORM that both report only a threefold expansion, which can be attributed to the needed photoswitching buffers. Thus, for some research, using SExY as an ExPALM method might diminish the need for additional expansion steps of the sample in iterative ExM protocols.

Possible future optimizations should be to implement a non-radical, highly defined lattice-like gel into the method, as recently introduced by the Tetragel [33,80]. This might lower loss of FP signal during gelation. Second, the range of suitable FPs should be extended. As the selection of FPs suitable for high-quality PALM imaging is rather small [2,40], we believe that engineering optimized FP variants for correlative ExM approaches based on current FPs might be an effective strategy. Engineering FPs that are not further affected by ExM protocol steps (e.g. that are less sensitive to a specific protein digestion method) offers a significant advantage [81]. This strategy has been shown successful for other correlative methods (e.g. in the development of mEos4 FPs). Here, nucleophilic amino acid residues that cross-link with aldehydes were replaced so that mEos4 variants tolerate osmium (OsO4) fixation—a treatment needed for electron microscopy [82]. Third, microscopes should be set-up for imaging at 50–80 µm depths to limit losses in localization precision that run counter to the gain in resolution provided by sample expansion.

Overall, the new correlative protocol is valuable for all yeast researchers who wish to combine ExM imaging with super-resolution imaging. In particular, we believe that the optimizations in protein retention yield, encompassing retention yield for fluorescent proteins, will also be of interest to a larger audience in microbial cell biology research and will extend to even more distant research areas if their interest involves studying low abundance proteins or for any reason relies on genetic labelling, e.g. when proteins are not readily accessible by extrinsic staining.

## Data Availability

Raw image data can be obtained from the authors on request. Custom relative frame-to-frame drift correction code is added as supplementary code and provided in the electronic supplementary material [83] as well as on github https://github.com/Endesfelder-Lab/frametoframedrift.
